# Assessment and Surgical Treatment of Calcinosis of the Shoulder Associated with CREST Syndrome

**DOI:** 10.1155/2016/9759182

**Published:** 2016-06-29

**Authors:** R. Manohara, S. J. Breusch

**Affiliations:** Edinburgh Royal Infirmary, Orthopaedic Department, 51 Little France Crescent, Old Dalkeith Road, Edinburgh, Midlothian EH16 4SA, UK

## Abstract

We report an unusual case of a 65-year-old lady with CREST syndrome with multiple upper and lower limb calcinosis, who presented with severe shoulder pain and stiffness, with widespread intra- and extra-articular calcinosis, which was refractory to conservative measures. We were able to identify the main cause of her symptoms through serial diagnostic injections as calcific biceps tendinosis. We will discuss her assessment and surgical management and the pathophysiology and various treatment modalities for managing the soft tissue calcinosis in rheumatological diseases.

## 1. Introduction

CREST syndrome is a limited form of scleroderma, characterised by calcinosis, Raynaud's phenomenon, esophageal dysmotility, sclerodactyly, and telangiectasia. The calcinosis is a particularly difficult entity to treat, given the paucity of effective treatment options in the literature. It is clinically often difficult to determine whether the calcinosis, irrespective of its manifestation intra- or extra-articularly, is the source for the patient's symptoms. The presentation varies from soft tissue swelling with associated pressure symptoms (e.g., with shoe wear), spontaneous discharge and infection, tendon infiltration with associated tendinopathy, or spontaneous tendon rupture or joint related pain. Clinical assessment and decision making can be challenging. We discuss a patient who presented with debilitating shoulder pain and stiffness for many years, who was successfully treated with surgery.

## 2. Case Report

Our patient first presented to us in late 2007. At that time, she was 65 years old, with a significant past medical history of CREST syndrome. She also had overlapping rheumatoid arthritis, for which she was on methotrexate. In addition, she had primary hypertension, left ventricular hypertrophy, and evidence of lung fibrosis on her CT thorax. Her main orthopaedic issue was that of debilitating right dominant shoulder pain and stiffness. This had compromised her already impaired day to day function as she had developed bilateral end stage sclerodactyly. She was unable to reach her buttock or hair and required much assistance for her activities of daily living. Her left shoulder was also affected, but to a less painful degree, and she also had developed other sites of symptomatic (ankle) and asymptomatic calcinosis.

On examination, she had multiple soft tissue calcific deposits visible on both hands and feet. She had end stage sclerodactyly with ankylosis of all her proximal and distal interphalangeal joints. She had petechial telangiectasis of her palms, mild clubbing of her fingers, and overall very poor hand function. Both shoulders had a passive forward flexion and abduction of 40 degrees and internal rotation to her buttock. External rotation was 15 degrees on the left side and −10 degrees on the right side. Rotator cuff testing was difficult due to limited movement and pain, but rotation against resistance produced pain. Active forward flexion produced pain and was severely limited to the extent that self-feeding was no longer possible. The severe pain on her right side was localised to the anterior shoulder region. Biceps tendon stress tests were inconclusive at the initial assessment. However, a rotator cuff pathology and intra-articular source of pain could not be excluded clinically.

**Figure 1:**
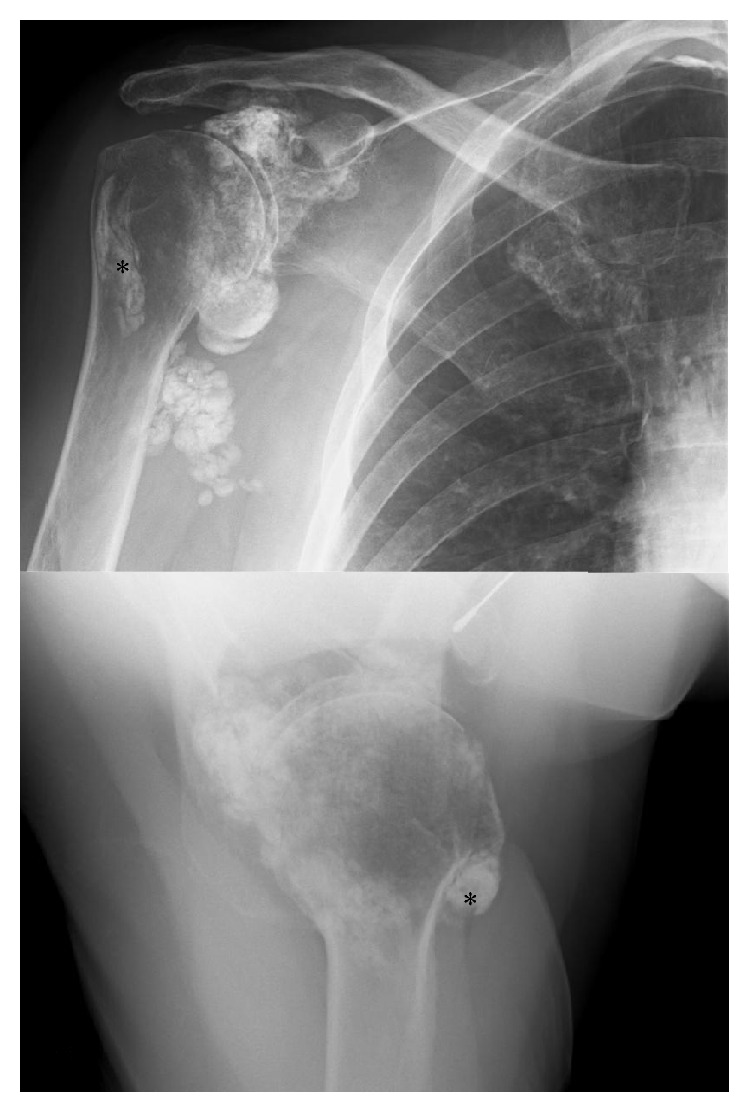
AP and axillary views of the right shoulder show widespread intra- and extra-articular calcinosis. Note the calcified biceps tendon in the bicipital groove, marked by an *∗*.

**Figure 2:**
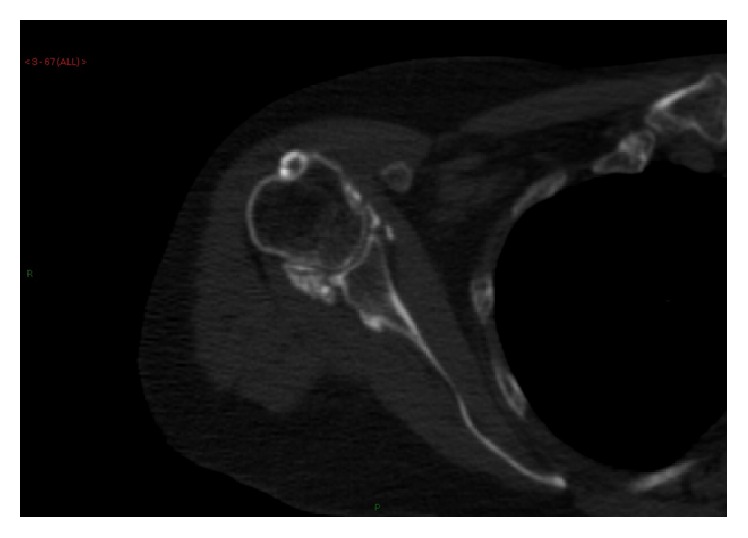
CT scan of the right shoulder confirmed intra- and extra-articular calcinosis.

**Figure 3:**
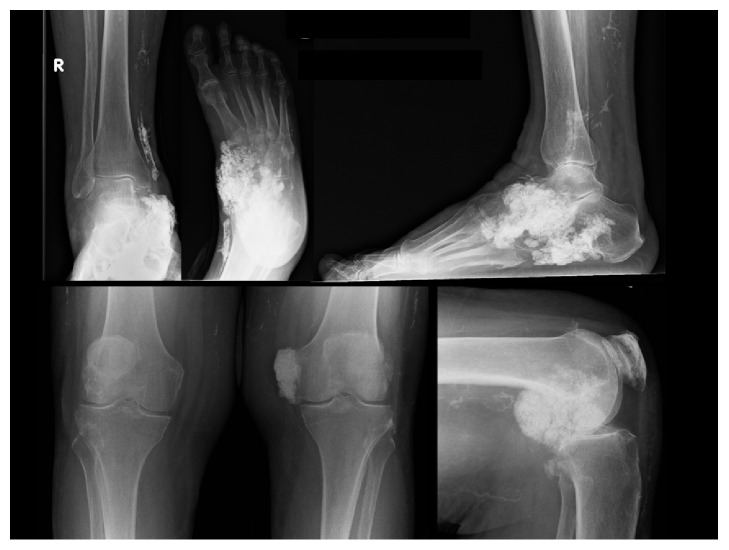
AP and lateral views of her right ankle and left knee show multiple manifestations and joint involvement of her calcinosis.

Her shoulder radiographs showed widespread calcinosis in the periarticular soft tissues but also suspected intra-articular infiltration (Figure 1). She was given a local steroid/anaesthetic injection to her intra-articular space and referred to physiotherapy and acupuncture. A CT scan was requested which showed no evidence of joint space narrowing or erosive arthropathy but confirmed both intra- and extra-articular calcinosis (Figure 2). Incidentally, she was also noted to have significant calcinosis in her right ankle and left knee joints as well (Figure 3).

At her next review, she reported that the injection had given her no relief at all and that her symptoms and presentation remained largely unchanged. Repeat examination confirmed persistent mainly anterior shoulder pain and a positive Yergason's biceps tendon stress test was evident. Given the localised pain and calcific mass in the region of the bicipital groove, which was evident on the radiographs and confirmed on the CT scan, she was given another local steroid/anaesthetic injection to the biceps tendon. The infiltration led to instant pain relief and some improved movement in clinic, and she was advised to continue on with her physiotherapy.

Over the next year, her symptoms and pain fluctuated and she was given 2 further injections with some prolonged benefit. Self-feeding was restored and she would on rare occasions enjoy “good” function and was able to put on her jacket and do her toileting independently. Her case was discussed with several local and external shoulder specialists. The unanimous opinion was to avoid open debridement surgery due to the extensive nature of the calcinosis and the worry that the capsulotomy performed would give rise to a marked collagenosis response which would lead to increased stiffness and poorer function ultimately. Eventually, should she develop joint arthropathy, shoulder arthroplasty could be a consideration. She was hence given repeat local steroid/anaesthetic injections to her biceps tendon as required and continued with physiotherapy for the time being.

However, by late 2009, her right shoulder pain and stiffness had become constant, unresponsive to injections (with only short term benefit), and significant enough to revisit the indication for surgery. She was counselled regarding the possible risks of infection, failure of the procedure to alleviate symptoms, increased stiffness, and possible recurrence of symptoms.

She underwent an open exploration of her right shoulder joint, excision of calcific deposits, and biceps tenodesis in November 2009. This was done under general anaesthetic with an interscalene block and antibiotic prophylaxis. Through a small 5 cm skin incision along the deltopectoral approach, the shoulder joint was approached. The subscapularis was incised and reflected and a capsulotomy was made. A chalky white stained effusion was drained from the joint. The shoulder was not dislocated, but it was inspected and found to have no intra-articular loose bodies. A thorough washout was performed. The biceps tendon was then explored and found to have a degenerative rupture, with massive, hard solid calcific deposits. These were excised to the level of healthy normal tendon, which was then tenodesed with a suture anchor below the bicipital groove. After further washout, the subscapularis was repaired, allowing for neutral rotation. The wound was closed in layers, and she was referred to early postoperative physio (limiting external rotation) for mobilisation.

At her clinical reviews at 3 months and 18 months postoperatively, she was extremely pleased with the continued pain relief from the procedure. Her active range of movement in the shoulder was also much better, and she was able to reach her mouth and feed herself. Her surgical wound had healed well and she had no residual tenderness over the anterior aspect of her proximal humerus. At a most recent follow-up in 2016 her status quo in relation to her right shoulder had been maintained with no decline in function and no recurrence of shoulder pain.

## 3. Discussion

In 1910, Thibierge and Weissenbach described the first case report of what was later called CRST (calcinosis cutis, Raynaud phenomenon, sclerodactyly, and telangiectasia) syndrome by Winterbauer in 1964. Although Winterbauer noted esophageal dysmotility in 4 of the 8 patients in his series, he did not include this feature in his original description of CRST syndrome. Frayha et al. suggested the acronym CREST in 1973 to include this frequently associated feature. Other manifestations of the disease generally are rare but include arthralgias, myopathy, pulmonary hypertension, myocardial involvement, primary biliary cirrhosis, renal crises, GI tract autonomic dysfunction, entrapment neurologic syndromes, and an increased risk of cancer, in particular lung cancer. CREST syndrome has been found to have a better prognosis than the systemic scleroderma types with diffuse skin involvement [1].

The calcinosis in CREST is due to accumulation of calcium apatite crystals, with normal levels of serum calcium, phosphorus, and alkaline phosphatase. The incidence of calcinosis in patients with limited cutaneous scleroderma is approximately 44% [2]. The management of calcinosis is aimed at either stopping the progression of dystrophic calcium formation or debulking the established calcific deposits. This is done by pharmacological, surgical, and various other adjunctive treatment modalities.

The pharmacological options are vast. A low dose warfarin regimen (1 mg/day) appears to have no demonstrable adverse effects, with a few reports in the literature suggesting beneficial outcomes [3]. Colchicine, probenecid, diltiazem, aluminium hydroxide, minocycline, and bisphosphonates have also been used. However, there is no strong evidence in the literature to support their use or efficacy. Intravenous immunoglobulins have been found to be unreliable [1]. Topical intralesional corticosteroids have also been used with variable success.

Amongst the adjunct treatment modalities, Chamberlain and Walker have described seeing a remission of symptoms in cutaneous calcinosis linked to CREST syndrome using CO_2_ laser treatment [4]. There have also been some reports of successful calcinosis treatment in CREST syndrome using extracorporeal shockwave lithotripsy [5].

Indications for surgical excision usually include failed medical management and large deposits causing (neuropathic) pain, deformity, disability, skin ulceration, or erosive arthropathy. Surgery is not without its risks which include infection, wound breakdown, failure of surgery, risk of recurrence, and the occasional need for skin flap cover after debridement. In this case, we were able to find the root of her problem through serial diagnostic local anaesthetic injections, and both patient and surgeon were happy that they took the brave step to proceed with her surgery, which has resulted in a satisfactory outcome to date.

Although clearly no firm conclusions can be made from a single case, it is of note that no adverse consequences of increased shoulder stiffness resulted from the capsulotomy performed. Clinical assessment of a painful shoulder in the background of calcinosis and significant stiffness is difficult, but serial diagnostic injections have paved the way to an accurate diagnosis and successful surgical management. We hope that this report may help in the management of patients with CREST, who present with shoulder pain on top of stiffness.
